# Balanced electrolyte solution with 1% glucose as intraoperative maintenance fluid in infants: a prospective study of glucose, electrolyte, and acid–base homeostasis

**DOI:** 10.1016/j.bja.2024.08.041

**Published:** 2024-11-05

**Authors:** Ulf Lindestam, Åke Norberg, Peter Frykholm, Olav Rooyackers, Andreas Andersson, Urban Fläring

**Affiliations:** 1 Department of Paediatric Perioperative Medicine and Intensive Care, Astrid Lindgren Children's Hospital, Karolinska University Hospital, Stockholm, Sweden; 2 Department of Physiology and Pharmacology, Karolinska Institutet, Stockholm, Sweden; 3 Department of Clinical Interventions and Technology, Karolinska Institutet, Stockholm, Sweden; 4 Department of Perioperative Medicine and Intensive Care, Karolinska University Hospital, Stockholm, Sweden; 5 Department of Anaesthesia and Intensive Care, Section of Paediatric Anaesthesia and Intensive Care, Uppsala University Hospital, Uppsala, Sweden; 6 Department of Surgical Sciences, Section of Anaesthesiology and Intensive Care Medicine, Uppsala University, Uppsala, Sweden

**Keywords:** acid–base physiology, glucose, infants, ketone bodies, perioperative fluid management, sodium

## Abstract

**Background:**

Optimal composition and infusion rates of intravenous maintenance fluids for children undergoing surgery are not well defined. Avoidance of hypoglycaemia, ketosis, and hyponatraemia is important, and current guidelines recommend isotonic fluids containing 1.0–2.5% glucose. However, evidence for its safe use in infants is insufficient. The aim of this study was to investigate whether normoglycaemia is maintained in infants using a balanced electrolyte maintenance infusion with 1% glucose.

**Methods:**

Infants 1–12 months of age undergoing surgery were included in this prospective two-centre study. Intravenous maintenance fluid was given with infusion rates of 4–8 ml kg^−1^ h^−1^. Blood gas and ketone body analysis were performed at induction and at the end of anaesthesia. Plasma glucose concentration was monitored intraoperatively.

**Results:**

For the 365 infants included in this study, the median infusion rate of maintenance fluid was 3.97 (interquartile range 3.21–5.35) ml kg^−1^ h^−1^. Mean plasma glucose concentration increased from 5.3 mM at induction to 6.1 mM at the end of anaesthesia (mean difference 0.8 mM; 95% confidence interval 0.6–0.9, *P*<0.001). No cases of hypoglycaemia (<3.0 mM) occurred. Mean sodium concentration remained stable during anaesthesia. Chloride and ketone body concentration increased and base excess decreased, but these were within the normal range.

**Conclusions:**

In infants undergoing surgery, maintenance infusion with a balanced electrolyte solution containing 1% glucose, at rates similar to those proposed by Holliday and Segar is a safe alternative with regards to homeostasis of glucose, electrolytes, and acid–base balance.

**Clinical trial registration:**

ACTRN12619000833167.

**Editor's key points**

• Optimal compositions and infusion rates of intravenous maintenance fluids for children undergoing surgery to avoid hypoglycaemia, ketosis, and hyponatraemia are have not been established.
• This two-centre prospective observational study investigated maintenance of normoglycaemia using a balanced electrolyte maintenance infusion with 1% glucose in infants undergoing surgery.
• Use of a balanced electrolyte maintenance fluid with 1% glucose was not associated with hypoglycaemia or electrolyte abnormalities intraoperatively in infants, even during prolonged anaesthesia (>4 h).

As emphasised in the expert initiative Safe Anesthesia For Every Tot (www.safetots.org), as well as in international guidelines for paediatric anaesthesia, the aim of intraoperative fluid therapy should be to maintain or re-establish normal physiology with regards to metabolic, electrolyte, and acid–base balance.^1^ In particular, hypoglycaemia is of great concern since, if severe and untreated, it affects cerebral perfusion and metabolism and can result in permanent neurological damage.^2^ Infants have a greater risk of developing intraoperative hypoglycaemia because of their higher metabolic rate and lower energy stores compared with older children and adults.^3^

Prolonged fasting before and during anaesthesia induces hepatic glycogenolysis. When this source of glucose is depleted, metabolism shifts to lipolysis and beta-oxidation with production of ketone bodies as alternative energy substrates. This protects the central nervous system from energy deficiency, but the negatively charged ketone bodies can lead to metabolic acidosis.^4^ However, excessive glucose supply can induce perioperative hyperglycaemia which can aggravate global or focal cerebral ischaemia and impair the immune system, with potential negative postoperative outcomes.^5^, ^6^, ^7^, ^8^

Hyponatraemia can lead to severe complications as it will increase the influx of water into brain cells, causing intracellular cerebral oedema with severe neurological damage and death as possible consequences.^9^ The main mechanism causing hyponatraemia is increased secretion of arginine vasopressin (AVP) as a result of perioperative non-osmotic stimuli—such as pain, nausea, and surgical stress—that reduce the excretion of free water.^10^^,^^11^ The choice of intraoperative and postoperative intravenous fluid and infusion rates also influences the risk for hyponatraemia.

The glucose and sodium content of perioperative intravenous fluids used in children has been debated for decades. Previously, hypotonic solutions with 5–10% glucose were used owing to concerns about hypoglycaemia, but these have been abandoned because of the risk of hyponatraemia and hyperglycaemia. Current guidelines recommend balanced electrolyte solutions containing 1.0–2.5% glucose as intraoperative maintenance for children.^12^^,^^13^ The safety data for the commercially available balanced electrolyte solution containing 1% glucose is based on results from small trials in young children 0–4 years old.^14^ These older studies have limitations, including that few infants were studied, anaesthesia time was relatively short, and infusion rates of maintenance fluid were high (≥10 ml kg^−1^ h^−1^).^15^, ^16^, ^17^ Moreover, the solutions used in these studies were different. Some were closer to normal osmolality with acetate and malate as bicarbonate precursors—for example, Sterofundin (B.Braun, Milan, Italy; Na^+^ 145 mM)—whereas others were hypotonic with lactate as bicarbonate precursor (Polyionique B66, Paris, France; Ringer's lactate with dextrose 1%, Na^+^ 120–130 mM). In current anaesthetic practice in Sweden, lower infusion rates of maintenance fluid are normally used, regional and neuraxial blocks are common, and fasting times have been reduced. These factors affect the risk of perioperative hypoglycaemia in different and sometimes opposing ways, and new data verifying the safety of 1% glucose solutions in this setting are needed.

The primary aim of this study was to evaluate the incidence of hypoglycaemia when a balanced electrolyte solution containing 1% glucose is used intraoperatively as a maintenance infusion to infants (1–12 months of age) in anaesthetic practice, with lower infusion rates of maintenance fluid, shorter fasting times, and frequent use of neuraxial blocks. Secondary aims were to evaluate the incidences of hyponatraemia and hyperchloraemia, increased ketone body concentration, metabolic acidosis, and the influence of prolonged duration of preoperative fasting.

## Methods

This prospective observational study was conducted at the Department of Paediatric Anaesthesia and Intensive Care at Astrid Lindgren Children's Hospital, Karolinska University Hospital (Stockholm, Sweden) and at the Paediatric Section of the Department of Anaesthesia and Intensive Care of Uppsala University Hospital (Uppsala, Sweden), both tertiary referral centres for paediatric surgery. Patients were included from September 2019 to November 2023. Inclusion criteria were children with body weight >4 kg, aged 28 days to 12 months, undergoing surgery with a predicted duration >30 min under general anaesthesia. Exclusion criteria were prematurity with postconception age <44 weeks, metabolic or endocrine disease, liver disease affecting liver function, malnutrition, growth retardation (>2 standard deviations, SD), ongoing beta-blocker therapy, preoperative infusion of parenteral nutrition or glucose >6 h, and plasma glucose concentration <3.0 mM at induction of anaesthesia. The exclusion criteria represent mainly groups of patients who exhibit special risk factors for intraoperative hypoglycaemia and are likely to have higher intraoperative glucose requirements. The study protocol was approved by the Swedish Ethical Review Authority (reference no. 2019-01153), and was registered at the Australian New Zealand Clinical Trials Registry (ACTRN12619000833167).

Parents were invited to participate at the preanaesthesia assessment or on the day of the operation in the surgical ward. They were presented with formalised oral and written study information, and written consent was obtained prior to inclusion.

The choice of induction and maintenance of anaesthesia was left to the discretion of the anaesthetist. All infants received intravenous infusion of a balanced electrolyte solution containing 1% glucose (Benelyte, Fresenius Kabi, Uppsala, Sweden) (composition in Table 1). Infusion rates were at the anaesthetist's discretion but were mainly according to local guidelines, ranging from 4 ml kg^−1^ h^−1^ for minor surgery to 8 ml kg^−1^ h^−1^ for major surgery with a higher infusion rate the first hour (10 ml kg^−1^ h^−1^) to compensate for preoperative fasting. No other glucose-containing drugs or infusions were given during anaesthesia. Intravenous fluid boluses with Ringer's acetate, albumin 5%, or blood products were given at the anaesthetist's discretion. At the time of the study, fasting regulations at the participating institutions adhered to the 2018 consensus statement from European Paediatric Anaesthesia organisations^18^ stating 6 h for solids, 4 h for breastmilk/formula, and 1 h for clear fluids.

**Table 1:** Composition of the infusion solution used (Benelyte, Fresenius Kabi, Uppsala, Sweden). ∗Effective osmolarity without glucose.

Sodium (mM)	140
Potassium (mM)	4
Calcium (mM)	1
Magnesium (mM)	1
Chloride (mM)	118
Acetate (mM)	30
Glucose (mM)	55.5
Osmolarity∗ (mOsmol L^−1^)	294

Blood sampling was performed directly after induction of anaesthesia and at the end of anaesthesia. It included bedside blood gas analysis (ABL 90, Radiometer, Brønshøj, Denmark) where base excess (BE), haematocrit, and concentrations of plasma haemoglobin, sodium, chloride, glucose, and lactate were obtained. At the same time points, plasma ketone bodies (β-hydroxybutyrate) were measured separately (FreeStyle Precision Neo, Abbott Diabetes Care Ltd, Witney, UK). Blood samples were drawn from an arterial or central/peripheral venous line. In the majority of cases, another line—separate from the one used for maintenance infusion—was used for sampling. If samples were drawn from a line used for glucose-containing infusions, great care was taken to minimise contamination.

Patient age (months) and weight, fasting time for solids, breastmilk, or formula, and glucose-containing clear fluids were registered along with information about any preoperative glucose infusion, type of surgery, and whether regional anaesthesia was used. At the end of anaesthesia, total volumes of all fluids given intraoperatively, duration of anaesthesia, and volume of blood loss were registered. Administration of α_2_-adrenoceptor agonists (clonidine, dexmedetomidine) and corticosteroids was also noted.

In addition to the blood gas analyses, plasma glucose concentration was monitored during anaesthesia every 30 min until 120 min and hourly thereafter, according to clinical routines. Hypoglycaemia was defined as plasma glucose <3.0 mM, and hyperglycaemia was defined as mild at plasma glucose >6.1 mM and as significant at plasma glucose >8.3 mM.^19^

Other adverse events included hyponatraemia (<135 mM), increased ketone body concentration (≥0.6 mM), hyperchloraemia (>110 mM), metabolic acidosis (BE below –5 mM), and prolonged fasting for breastmilk or formula (>6 h). Simplified strong ion difference (SID) was calculated by subtracting chloride from sodium concentrations.

### Statistical analysis

Although uncommon events can never be completely ruled out, we wanted to investigate enough subjects so that in the case of zero events of hypoglycaemia we would reach a 95% confidence interval (95% CI) of 0–0.010 for the fraction of hypoglycaemia. According to the Wilson–Brown method, and assuming zero events, we determined a sample size of *n*=365 to be sufficient to meet this goal.

Visual inspection of histograms together with a summary for skewness were used to assess normality. Normally distributed data are presented as mean (SD), whereas differences are presented as mean (95% CI of the mean) and analysed by the paired Student *t*-test. For clarity, figures of the main variables are presented with median, interquartile range (IQR), 1 and 99 percentiles, and outliers in the form of boxplots.

Data not following a normal distribution are presented as median (IQR) and analysed by the Wilcoxon signed rank test. Discrete variables are presented as frequency (percentage) and compared by Fisher's exact test. *Post hoc* analyses between subgroups were performed by independent two-sample *t*-test, and presented as differences between means (95% CI) or Mann–Whitney *U*-test, as appropriate.

Two-sided *P*-values are reported, and a value <0.05 was considered significant. GraphPad Prism 10 was used for statistical analysis (GraphPad Software, Boston, MA, USA; www.graphpad.com). The STROBE Statement checklist for cohort studies was used when reporting this study.

## Results

**Fig 1:**
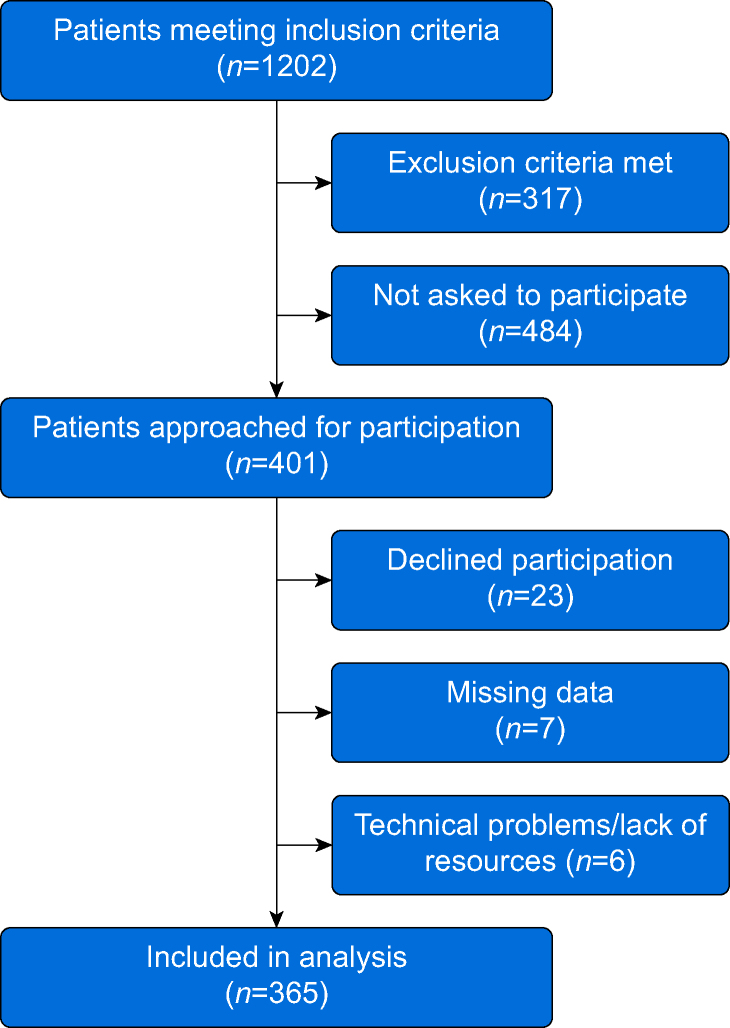
STROBE flow chart.

The total time for inclusion stretched over >4 years. Inclusion was interrupted for periods owing to lack of resources (e.g. research nurses), and the coronavirus disease 2019 (COVID-19) pandemic reduced the number of eligible patients. Fig. 1 describes the inclusion process; 365 patients were included in the study and in the final analyses.

Subject characteristics and details regarding perioperative fluid management are given in Table 2. Operations performed were abdominal in 35% of subjects, cleft lip palate in 16%, ophthalmic in 15%, urological in 10%, ear, nose, and throat in 5%, neurosurgical in 5%, orthopaedic in 5%, and other in 9%. The median intraoperative infusion rate of balanced electrolyte solution with 1% glucose was 3.97 (IQR 3.21–5.35) ml kg^−1^ h^−1^. Bolus fluid therapy with Ringer's acetate or albumin 50 mg ml^−1^ was administered in 119 patients (33%), whereof eight patients received boluses of both fluid types. Blood products were used in 12 cases (3%).

**Table 2:** Subject characteristics for 365 children undergoing surgery with maintenance infusion of 1% glucose. Normally distributed data are expressed as mean (SD) and data not following a normal distribution as median (interquartile range, IQR). Discrete variables or proportions are presented as *n* (%). ASA, American Society of Anesthesiologists.

Age (months), mean (range)	5.2 (1–12)
Weight (kg), mean (SD)	7.0 (1.8)
Male, *n* (%)	229 (63)
Duration of anaesthesia (min), median (IQR)	125 (90–200)
ASA physical status 1 or 2, *n* (%) ASA physical status 3, *n* (%)	305 (83.6) 60 (16.4)
Regional anaesthesia, *n* (%) Neuraxial anaesthesia, *n* (%)	162 (44) 91 (25)
Fasting duration breastmilk or formula (h): median (IQR) Fasting duration clear fluids (h), *n*=49 (13.4%): median (IQR) Fasting duration solids (h), *n*=20 (5.5%): median (IQR)	5 (4–7) 2 (1.4–3.0) 11 (8–13)
Maintenance infusion 1% glucose (ml kg^−1^ h^−1^): median (IQR)	3.97 (3.21–5.35)
Bolus fluid Ringer's acetate (ml kg^−1^), *n*=28, (7.7%): median (IQR) Bolus fluid albumin 5% (ml kg^−1^), *n*=99 (27%): median (IQR)	9.1 (4.9–10.0) 9.6 (5.8–10.2)
Blood products (ml kg^−1^), *n*=12 (3.3%): median (IQR)	10.2 (8.8–11.0)
Bleeding (ml) *n*=48 (13.2%): median (IQR)	20 (10–20)

**Fig 2:**
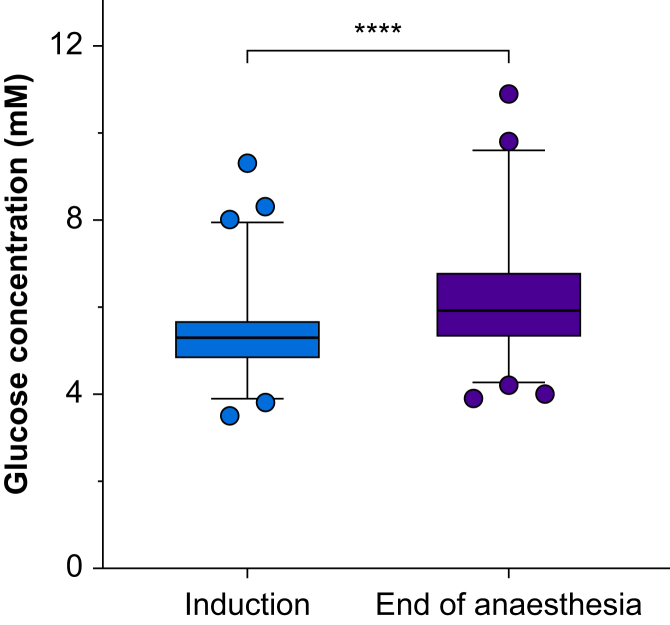
Plasma glucose concentrations at induction and conclusion of anaesthesia. Data expressed as box plots (boxes show median and interquartile range, whiskers show 1 and 99 percentiles). ∗∗∗∗ p < 0.0001.

**Figure S1:**
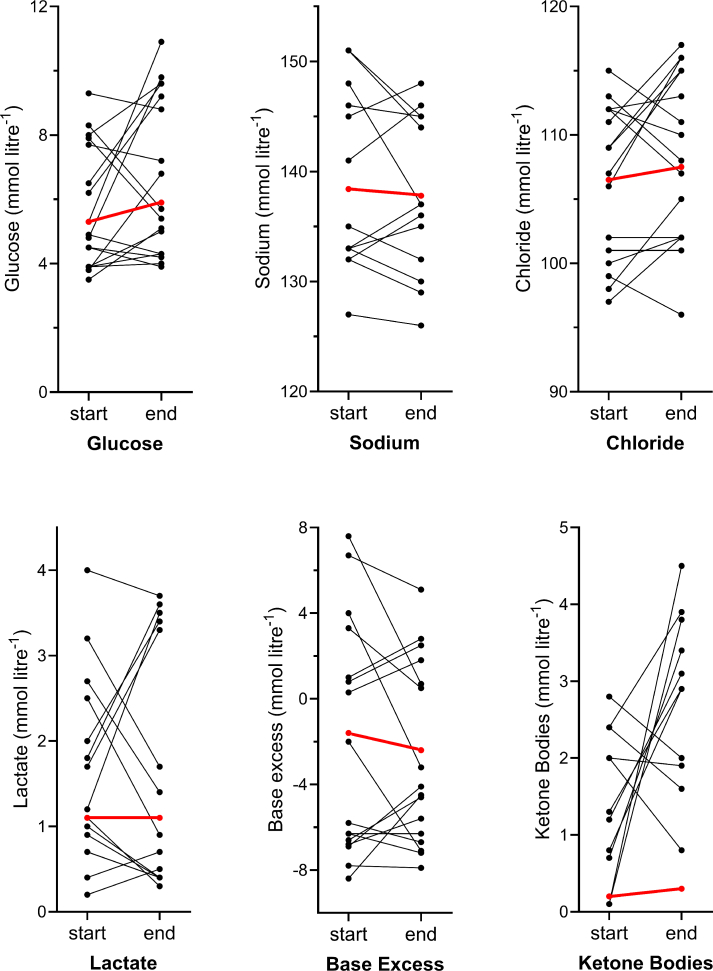
Outliers of acid-base variables and glucose, electrolyte and ketone body concentrations at the induction and at the end of anaesthesia in 365 children undergoing surgery. Data obtained for the 3–6 subjects with the lowest and highest concentrations at the start and end of anaesthesia, respectively. Red symbols and lines depict median values of all subjects.

Mean plasma glucose concentration increased from induction (5.3 mM) to the end of anaesthesia (6.1 mM) (mean increase 0.8 mM; 95% CI 0.6–0.9, *P*<0.001). The majority of the patients remained within the normal range (Fig. 2). No cases of hypoglycaemia (<3.0 mM) were found, including intraoperative measurements performed for safety reasons. The three patients with the lowest plasma glucose concentrations at the end of anaesthesia all had relatively low concentrations at induction (Supplementary Fig. S1). Hyperglycaemia (>8.3 mM) was found in one child at induction of anaesthesia and in 16 (4.3%) at the end of anaesthesia.

Children ≤3 months of age (*n*=123, 34%) had lower mean glucose concentrations at induction of anaesthesia (difference between means: 0.18 mM; 95% CI 0.01–0.30, *P*=0.033) and a larger mean intraoperative increase in glucose concentrations (difference between means 0.3 mM; 95% CI 0.1–0.6, *P*=0.005) compared with older infants.

**Fig 3:**
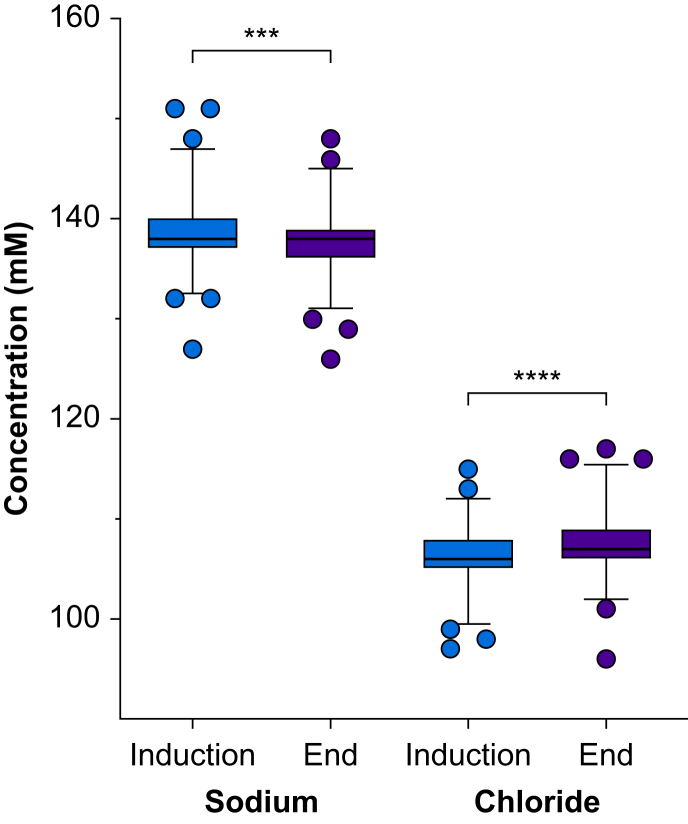
Plasma sodium and chloride concentrations at induction and conclusion of anaesthesia. Data expressed as box plots (boxes show median and interquartile range, whiskers show 1 and 99 percentiles). ∗∗∗ p < 0.001, ∗∗∗∗ p < 0.0001.

Mean plasma sodium concentration decreased throughout the intraoperative period within (or close to) the normal range (Fig. 3) (mean decrease 0.7 mM; 95% CI 0.4–0.9, *P*<0.001). The proportion of patients with hyponatraemia (<135 mM) was 3.6% at induction and 6.6% at the end of anaesthesia. The three patients with the lowest plasma sodium concentrations at the end of surgery were all hyponatraemic at induction (Supplementary Fig. S1). The mean change in plasma sodium concentration was smaller in those who received bolus fluid therapy with albumin 5% compared with those who did not (difference between means 0.8 mM; 95% CI 0.3–1.3, *P*=0.002).

Mean chloride concentration increased within the normal range and BE decreased (Table 3 and Supplementary Table S1). The proportion of subjects with hyperchloraemia (defined as plasma chloride concentration >110 mM) at the end of anaesthesia was higher in cases with duration of anaesthesia >180 min (*n*=103) as compared with shorter procedures (20% *vs* 10% respectively; *P*=0.020). In total, 4.7% of the subjects were hyperchloraemic (>110 mM) at induction as compared with 13.2% at the end of anaesthesia. At the same time points, the proportions of children with metabolic acidosis (BE below –5 mM) were 3.3% and 8.1%, respectively. In the group who received bolus fluid therapy with albumin 5%, the mean increase in chloride concentration was larger than in those who did not receive albumin 5%, but the difference was small (difference between means 1.0 mM; 95% CI 0.4–1.5, *P*<0.001).

**Table 3:** Acid–base variables and glucose, electrolyte, haemoglobin, and ketone body concentrations at the induction and at the end of anaesthesia in 365 children undergoing surgery. Descriptive data are expressed as mean (SD), or median (IQR), differences as mean (95% CI) or median (IQR). ^∗^One sample *t*-test; ^†^Wilcoxon signed rank test.

	Induction Mean (SD)	End Mean (SD)	Mean difference (95% CI)	*P*-value
Glucose (mM)	5.3 (0.8)	6.1 (1.1)	0.8 (0.6–0.9)	<0.001^∗^
Sodium (mM)	138 (3)	138 (3)	–0.7 (–0.9 to –0.4)	<0.001^∗^
Chloride (mM)	107 (2)	108 (3)	1.0 (0.7–1.3)	<0.001^∗^
Calcium (mM)	1.34 (0.5)	1.31 (0.6)	–0.04 (–0.04 to –0.03)	<0.001^∗^
Haemoglobin (g L^−1^)	109 (12)	99 (11)	–10 (–11 to –9)	<0.001^∗^
Haematocrit (%)	33 (4)	30 (3)	–3 (–3.4 to –2.9)	<0.001^∗^
Base excess (mM)	–1.5 (1.9)	–2.4 (1.8)	–0.8 (–1.0 to –0.6)	<0.001^∗^
Strong ion difference (mM)	31.9 (2.6)	30.2 (3.0)	–1.6 (–1.9 to –1.4)	<0.001^∗^
Lactate (mM)	1.0 (0.8–1.2)	1.0 (0.8–1.3)	0.0 (–0.2 to 0.3)	0.17^†^
Ketone bodies (mM)	0.2 (0.1–0.4)	0.4 (0.2–0.6)	0 (–0.1 to 0.3)	<0.001^†^

Median ketone concentration increased from induction to the end of anaesthesia—0.2 mM (IQR 0.12–0.4) and 0.3 mM (IQR 0.2–0.6), respectively—corresponding to a median difference of 0.0 (IQR –0.1 to 0.3, *P*<0.001). The increase was higher in patients with duration of anaesthesia >180 min compared with shorter procedures (0.3 mM; IQR 0.0–0.7 *vs* 0.0 mM; IQR –0.1 to 0.1) (*P*<0.001)). The proportion of subjects with ketone body concentration ≥0.6 mM was 15.7% at induction and 30.6% at the end of anaesthesia. Mean BE at induction of anaesthesia was lower in children with ketone body concentration ≥0.6 mM compared with those with normal ketone body concentrations (–2.8 mM and –1.4 mM, respectively, difference between means 1.4 mM; 95% CI 0.9–1.9, *P*<0.001). This difference remained at the end of anaesthesia.

Information on fasting time for solids was available in 20 subjects, all of whom were older than 6 months. Median fasting time for breastmilk or formula was 5 h (IQR 4–7). Forty-five subjects (12.3%) received a glucose-containing clear drink <4 h before anaesthesia. No information on volume of intake or caloric content was available. There were no significant differences in subjects with fasting times >6 h compared with shorter fasting times regarding preoperative and postoperative glucose and ketone body concentrations. The proportion of subjects with prolonged fasting times (>6 h) for breastmilk or formula or clear fluids was 21% (73 out of 355 with complete data).

Corticosteroids (mainly betamethasone) were administered to 86 subjects (23%). Subjects who received corticosteroids had a higher mean intraoperative increase in glucose concentration compared with those not receiving corticosteroids (difference between means 0.4 mM; 95% CI 0.1–0.7, *P*=0.005). However, there was no difference between these groups regarding the proportion of patients with hyperglycaemia at the end of anaesthesia. Clonidine was administered to 340 subjects (93%) either orally before anaesthesia, intravenously as part of multimodal pain management, or as an adjuvant in regional anaesthesia blocks. Comparing these subjects with those who did not receive clonidine, no difference regarding perioperative change in plasma glucose concentrations was seen (difference between means 0.0 mM; 95% CI –0.4 to 0.5, *P*=0.841).

There were 162 subjects who received regional anaesthesia preoperatively. When comparing these subjects with those who did not receive regional anaesthesia, no difference was seen in the change in plasma glucose from induction to the end of anaesthesia. Similarly, there was no difference in intraoperative glucose change when further analysing those who received a neuraxial block (*n*=91, epidural 11, caudal 80) compared with those who did not.

## Discussion

This large prospective observational study provides evidence that balanced electrolyte maintenance fluid with 1% glucose is not associated with hypoglycaemia intraoperatively in infants without risk factors for hypoglycaemia, even during prolonged anaesthesia (>4 h). Median plasma glucose increased by 11%, and even more among the youngest infants (≤3 months of age). Electrolyte concentrations were quite stable, and we observed a slight trend towards decreasing BE without clinical significance. The incidence of supranormal ketone body concentration increased, and the proportion of subjects with acidosis (although mild) increased slightly. Fasting times for breastmilk or formula showed acceptable adherence to current guidelines, but 21% of children had fasted for >6 h preoperatively.

Hypoglycaemia is a rare complication in paediatric anaesthesia, but it can have devastating consequences. When reviewing safety data regarding intraoperative maintenance fluid, both the type of solution and the infusion rate affect how much glucose is administered and consequently the risk for hypoglycaemia. In recent years, intraoperative fluid therapy has moved away from the use of high continuous rates of maintenance fluid towards lower maintenance infusion rates with additional boluses of fluid to match deficits caused by to losses during surgery.^20^ In our subjects, the median infusion rate of balanced electrolyte maintenance fluid with 1% glucose was 4 ml kg^−1^ h^−1^, which is similar to the recommended hourly fluid requirements in this age group.^21^ Previous safety data for 1% glucose solutions are based on studies using higher intraoperative maintenance infusion rates of ≥10 ml kg^−1^ h^−1^, calling into question whether these data are still valid. Moreover, the shorter fasting times and more frequent use of regional anaesthesia in modern paediatric anaesthesia could potentially affect the risk of intraoperative hypoglycaemia compared with older studies. Another possible concern with previous data is that only small numbers of infants were included, and that anaesthesia times were shorter compared with those of the current study. All these factors contribute to the need for more robust safety data for the use of 1% glucose solutions. We present prospective data from a large cohort of infants demonstrating the safety of a balanced electrolyte solution with 1% glucose as the intraoperative maintenance fluid for infants in the contemporary anaesthetic setting, even during extended periods of anaesthesia.

Possible explanations for stable glucose concentration during anaesthesia with only limited glucose administration are likely multifactorial. Infants from 1 month of age withstand starvation better than newborns and preterm infants.^3^^,^^22^ Furthermore, surgical stress triggers insulin resistance and endogenous glucose production, and anaesthesia decreases energy requirements.^23^, ^24^, ^25^ Regional anaesthesia, certain drugs (corticosteroids, clonidine), and fasting are also likely to affect perioperative glucose concentrations.

Despite the low glucose content and delivery in our study, mild hyperglycaemia (6.1–8.3 mM) was seen in 41% of the subjects. Only 4% of subjects (*n*=16) had a plasma glucose concentration >8.3 mM. If a 2.5% glucose solution had been used, it is likely that the incidence of hyperglycaemia would have been higher. Previous reports have shown that 2.5% glucose solutions in infants 1–12 months of age result in hyperglycaemia in up to 10% of cases, suggesting that solutions with 1% glucose are a better alternative for this age group.^26^

In general, plasma sodium concentration was maintained from induction to the end of anaesthesia. Only a minor decrease in plasma sodium concentration was observed, in concordance with previous randomised paediatric perioperative studies which showed a reduced incidence of hyponatraemia when near isotonic perioperative maintenance fluid was given as compared with hypotonic solution.^27^, ^28^, ^29^ However, these studies did not focus on intraoperative sodium concentrations, but rather on the postoperative phase. Near isotonic maintenance solutions are not always commercially available. In many countries, 2.5% glucose solutions are often hypotonic (70 mM Na^+^), making them a less favourable option in the setting of surgery as a substantial amount of free water will be given in combination with increased AVP secretion.^30^, ^31^, ^32^, ^33^ Our institutional routine has been to increase tonicity by adding extra sodium to this hypotonic solution (8.75 ml Addex-NaCl 4 mM to 500 ml), but this practice is not without risk of prescription and calculation errors, contamination, etc. Another option would be to add glucose to a balanced electrolyte solution (6 ml of 40% glucose in 250 ml increases glucose concentration by 1%).^13^ Subjects given bolus fluid therapy with albumin 5% exhibited smaller decreases in plasma sodium concentration and a larger increase in plasma chloride concentration compared with those who did not receive albumin 5%, most likely because of the hypertonic character of this solution. However, differences were small and without clinical relevance.

Acid–base changes from induction to the end of surgery were minor. There was only a tendency for increasing plasma chloride concentrations, decreasing BE, and SID. The maintenance fluid used has a relatively high chloride content (118 mM), and hyperchloraemia was more frequent in subjects undergoing procedures longer than 3 h. Hyperchloraemia, besides having an effect on decreasing SID and BE, can reduce renal blood flow.^17^ Therefore, risk of iatrogenic hyperchloraemia and metabolic acidosis should be kept in mind, especially when duration of surgery is prolonged.

Prolonged preoperative fasting can also influence intraoperative glucose concentration and result in increased blood ketone body concentration.^34^ However, our results showed no difference in either glucose or ketone body concentrations with regards to duration of fasting at induction or at the end of anaesthesia. Possible explanations are the generally short fasting times, maintained endogenous glucose production, and lower energy expenditure. The concentration of ketone bodies was within the normal range at the end of anaesthesia. Our results show that adherence to modern fasting guidelines is acceptable in this study, with median fasting times for breastmilk or formula of 5 h and a proportion of prolonged fasting times (>6 h) in 21% of subjects, which is lower than those in previous studies.^35^, ^36^, ^37^ The proportion of infants who received glucose-containing clear drinks <4 h before surgery was 12.3%. Possibly more patients could have received clear drinks preoperatively, but breastmilk or formula is often the only feed for infants, and this issue remains unresolved.

The majority of the subjects received either local anaesthetics as topical application (ophthalmic or ENT surgery), as infiltration, or as regional or neuraxial blocks. This would have increased the risk of hypoglycaemia as regional anaesthesia might reduce surgical stress.^15^^,^^16^^,^^38^ Our results suggest that the increased glucose production induced by surgical stress combined with the exogenous administration of a small dose of glucose overrides possible effects of regional anaesthesia.

Corticosteroids were used for their general anti-inflammatory properties or as a part of multimodal perioperative pain management in 24% of the subjects. Because of their hyperglycaemic properties, an increase in plasma glucose concentration would be expected, but the results showed only a small difference at the end of anaesthesia. Clonidine, an α_2_-adrenoceptor agonist associated with mild hyperglycaemic effects, might have influenced the results as it was used in 93% of subjects.

The limitations of this study are mostly related to its design, which included only two participating centres, and that inclusion was non-consecutive. In addition, total intravenous fluid volume—including flush, anaesthetic drugs, paracetamol, and antibiotics given intraoperatively—was not registered. Compared with the total, this added volume was minor and would not alter the final results of the study. The lack of strict rules for maintenance infusion rates can be viewed as a limitation. However, the study design reflects the clinical reality where the choice of initial and continued infusion rates is based on many variables (e.g. duration of fasting, age of the patient, and magnitude of the surgery) such that the final decision is left to the discretion of the anaesthetic team.

Strengths of the study include its prospective design and large number of subjects undergoing a variety of surgical procedures, with few missing data. Important variables involved in perioperative glucose metabolism, such as duration of preoperative fasting and ketone body concentration, are well characterised in a defined age cohort where information up to now has been lacking. In addition, the present study provides information specifically on intraoperative sodium concentration which is scarce in previous studies.

In conclusion, this study shows that in infants undergoing surgery, a balanced electrolyte solution containing 1% glucose as intravenous maintenance infusion at rates similar to those proposed by Holliday and Segar is a safe alternative regarding intraoperative homeostasis of glucose, sodium, and acid–base balance. However, since occurrence of asymptomatic preoperative or intraoperative hypoglycaemia cannot be excluded in rare cases, plasma glucose and sodium should be checked at induction and during anaesthesia in infants, especially during prolonged anaesthesia (>2 h).

## Authors' contributions

Study concept and design: UF, AA, UL, ÅN, OR

Data collection: PF, UL, UF

Data analysis: UL, UF, ÅN

Data interpretation: all authors

Drafting of the manuscript: UL, UF, AA

Critical revision of the manuscript: all authors.

All authors approved the final paper to be published and agree to be accountable for all aspects of the work, ensuring that questions related to the accuracy or integrity of any part of the work are appropriately investigated and resolved.

## Acknowledgements

We thank all the staff at the Department of Paediatric Anaesthesia at Astrid Lindgren Children's Hospital, Karolinska University Hospital, and in particular the research nurses Johannes Meijers, Ann-Marie Fläring, Marie-Christin Johansson, Aziza Abdella Mussa, and Anna Alvarsson, and Nathalie Danielsson at Uppsala University Hospital, for their excellent help in conducting the study. We also thank Henrike Häbel, statistician at the Medical Statistics Unit, Department of Learning, Informatics, Management and Ethics, Karolinska Institutet for valuable statistical discussions.

## Declaration of interests

OR received consulting reimbursement from Fresenius-Kabi, Nestlé, and Baxter and has an institutional research grant from Fresenius-Kabi. The other authors declare that they have no conflicts of interest.

## Funding

Small project grants from The Samariten Foundation for Paediatric Research (UF) and The Swedish Society for Paediatric Anaesthesia and Intensive Care, SFBABI (UL). Project nr K2021-9491.
