# Supplementary material for: Balanced electrolyte solution with 1% glucose as intraoperative maintenance fluid in infants: a prospective study of glucose, electrolyte, and acid–base homeostasis
Source: Br J Anaesth. 2024 Nov 5;134(5):1432–9. doi: 10.1016/j.bja.2024.08.041 (PMC12106871; doi:10.1016/j.bja.2024.08.041)
Supplement: Multimedia component 1 [file mmc1.docx]

Figure legend fig S1.

***Figure S1.*** *Outliers of acid-base variables and glucose, electrolyte and ketone body concentrations at the induction and at the end of anaesthesia in 365 children undergoing surgery. Data obtained for the 3-6 subjects with the lowest and highest concentrations at the start and end of anaesthesia, respectively. Red symbols and lines depict median values of all subjects.*
