# Supplementary material for: Balanced electrolyte solution with 1% glucose as intraoperative maintenance fluid in infants: a prospective study of glucose, electrolyte, and acid–base homeostasis
Source: Br J Anaesth. 2024 Nov 5;134(5):1432–9. doi: 10.1016/j.bja.2024.08.041 (PMC12106871; doi:10.1016/j.bja.2024.08.041)
Supplement: Multimedia component 2 [file mmc2.docx]

|  | **Induction**  median (range) | **End**  median (range) | **Difference** median (range) |
| --- | --- | --- | --- |
| Glucose  (mM) | 5.3 (3.5 to 9.3) | 5.9 (3.9 to 10.9) | 0.6 (-2.6 to 5.6) |
| Sodium  (mM) | 138 (127 to 151) | 138 (126 to 148) | -1 (-11 to 8) |
| Chloride  (mM) | 106 (97 to 115) | 107 (96 to 117) | 1 (-5 to 11) |
| Lactate  (mM) | 1.0 (0.2 to 4.0) | 1.0 (0.3 to 3.7) | 0.0 (-1.6 to 2.3) |
| Calcium  (mM) | 1.35 (1.15 to 1.38) | 1.31 (1.11 to 1.57) | -0.04 (-0.25 to 0.15) |
| Haemoglobin  (g L^-1^) | 108 (72 to 161) | 97 (67 to 141) | -10 (-41 to 26) |
| Haematocrit  (%) | 33 (24 to 49) | 30 (21 to 43) | -3 (-12 to 7) |
| Base Excess  (mM) | -1.5 (-8.4 to 7.6) | -2.3 (-7.9 to 5.1) | -0.5 (-7.2 to 3.9) |
| Ketone bodies  (mM) | 0.2 (0.0 to 2.8) | 0.3 (0.0 to 4.5) | 0.0 (-1.2 to 4.4) |
| Strong Ion difference  (mM) | 32 (24 to 47) | 30 (23 to 42) | -2 (-10 to 8) |

***Table S1.*** *Acid-base variables and glucose, electrolyte, haemoglobin and ketone body concentrations at the induction and conclusion of anaesthesia in 365 children undergoing surgery. All data are expressed as median (range).*
